# Concurrent exercise improves insulin resistance and nonalcoholic fatty liver disease by upregulating PPAR-γ and genes involved in the beta-oxidation of fatty acids in ApoE-KO mice fed a high-fat diet

**DOI:** 10.1186/s12944-018-0933-z

**Published:** 2019-01-05

**Authors:** Fan Zheng, Ying Cai

**Affiliations:** Cardiac Rehabilitation Center of Rehabilitation Department, Xiangya Hospital at Central South University, Xiangya Road 87#, Changsha, Hunan People’s Republic of China

**Keywords:** Concurrent exercise, Insulin resistance, Non-alcoholic fatty liver disease, PPAR-γ, CPT-1, MCAD

## Abstract

**Objective:**

To emphasize the mechanism of concurrent exercise effect on lipid disorders in insulin resistance (IR) and nonalcoholic fatty liver disease (NAFLD).

**Materials and methods:**

Twenty male ApoE knockout mice were randomly divided into two groups: HFD group (*n* = 10) fed a high fat diet, and HFDE group (*n* = 10) with high-fat diet intervention for 12 weeks and swimming exercise. Other ten healthy male C57BL/6 J mice were fed a normal diet, and included as control group. Retro-orbital blood samples were collected for biochemical analysis. Oil red O staining of liver tissues was performed to confirm the exercise effect. Western blotting was performed to evaluate the expressions of PPAR-γ, CPT-1, MCAD.

**Results:**

The levels of TG, TC, LDL, FFA, FIN, FPG and Homa-IRI in the HFD group were significantly higher than ND group, while these were markedly decreased in the HFDE group compared with HFD group. The Oil Red O staining of liver samples further confirmed the exercise effect on the change of lipid deposition in the liver. Western blotting showed increased expressions of PPAR-γ, CPT-1, MCAD induced by high fat diet were significantly downregulated by exercise.

**Conclusion:**

A concurrent 12-week exercise protocol alleviated the lipid metabolism disorders of IR and NAFLD, probably via PPAR-γ/CPT-1/MCAD signaling.

## Introduction

Type 2 diabetes mellitus (T2DM) and nonalcoholic fatty liver disease (NAFLD) are pandemic metabolic diseases. They act in a “vicious circle” that accelerates worsening of the vascular complications of T2DM and aggravates NAFLD progression to liver cirrhosis and hepatocellular carcinoma [1]. In humans, T2DM is one of the most common chronic diseases with increasing incidence due to lifestyle changes. It is characterized by hyperglycemia mainly caused by impaired insulin secretion and insulin resistance [2]. Insulin resistance (IR) undoubtedly plays a vital role in the pathogenesis of T2DM and other metabolic disorders, such as obesity, high blood pressure, high blood cholesterol and metabolic syndrome. Clinically, IR is strongly associated with stress, visceral adiposity, and declined cardiorespiratory fitness; also it increases the risk of cardiovascular disease. Biologically, IR is caused by elevated concentrations of free fatty acids (FFAs). Owing to excess fat intake, FFAs are deposited as triglycerides (TG) in non-fat tissues, e.g., skeletal muscle, liver, heart and pancreas [3, 4]. The impairment of fatty acid β-oxidation caused by IR leads to an abnormal accumulation of TG in the liver and the development of NAFLD. The most common independent risk factor for NAFLD is believed to be IR [5, 6]. Molecularly, IR is caused by an imbalance between excess nutrients or inflammatory cytokines and the cell membrane receptors [7, 8].

Meanwhile, NAFLD is defined as excess fat accumulation in the liver due to causes other than excess alcohol consumption [9]. It represents the most prevalent chronic liver disease in the world; nearly 70% of the overweight population has NAFLD [10]. Several studies demonstrated that NAFLD was not only closely linked to liver-related morbidity or mortality, but also to an increased risk of diabetes and cardiovascular disease [5, 11–14]. Studies further clarified that NAFLD was an independent risk factor for the development of T2DM [7]. Though NAFLD might be reversible in the early stages, it is hard to discover because of its asymptomatic nature. Along with IR, this chronic liver condition progressively worsens and is usually not recognized until the patients develop cirrhosis [15]. Therefore, it is vital to prevent and treat IR and NAFLD in the early stages.

The incidence of NAFLD is growing worldwide, because of both the rising tide of T2DM and obesity and the lack of effective treatments. Since IR is believed to be the main cause of NAFLD, drugs targeting IR may be used for the treatment of NAFLD. Some thiazolidinediones, primarily used to improve IR and treat T2DM, were proven to be effective in alleviating the hepatic steatosis and fibrosis in patients with NAFLD. However, there are few validated pharmacological interventions for NAFLD [16, 17]. The current management of NAFLD concentrates on lifestyle modification, weight loss, and exercise. Exercise has been proven to be a significant and powerful low-risk means of improving both IR [3] and NAFLD [18], but the underlying multiple mechanisms of managing the disease still remain largely unknown.

Problems with fat metabolism are associated with NAFLD. Peroxisome proliferator-activated receptors (PPAR) belong to the nuclear hormone receptor superfamily, and play a pivotal role in glucose and fatty acid metabolism. Three members of this superfamily have already been discovered: PPAR-α, PPAR-β/δ and PPAR-γ [19]. Since NAFLD is basically caused by an abnormal fat metabolism, these nuclear receptors are key modulators in the pathological course of the disease, and they are also candidate targets for treating NAFLD [20, 21]. Specifically, PPAR-γ, which is known as ‘energy-balanced receptor’, has been shown to be a crucial regulator in both IR [22–25] and NAFLD [26, 27]. This is because PPAR-γ regulates several downstream genes related to fatty acid oxidation, such as carnitine palmitoyl transferase-1 (CPT-1) [28] and medium-chain acyl-CoA dehydrogenase (MCAD) [29]. Significantly, CPT-1 initially catalyzes the transportation of fatty acids into the mitochondria for β-oxidation, followed by a further catalysis of fatty acid β-oxidation in the mitochondrial matrix by MCAD. In short, CPT-1 and MCAD are both considered to be key enzymes for lipid metabolism [25, 30, 31]. Hence, in this study, we hypothesized that exercise might ameliorate IR and NAFLD through PPAR-γ/ CPT-1/MCAD pathway regulation. To this end, we provided an experimental model of IR and NAFLD by using Apolipoprotein-E-knockout (ApoE-KO) mice, which were fed a high fat diet (HFD). ApoE is an important constituent of all plasma lipoproteins and serves as a ligand for cell-surface lipoprotein receptors such as the LDL-receptor. ApoE-KO mice spontaneously develop hypercholesterolemia and atherosclerosis when fed a standard chow. An HFD can further exacerbate these lesions and accelerate the process. Furthermore, studies established that ApoE-KO mice fed an HFD could serve as a rapid and valuable NAFLD model [32, 33]. Next, we explored the underling mechanism of the effect of exercise on IR and NAFLD by detecting PPAR-γ/ CPT-1/MCAD expressions in the liver of ApoE-KO mice fed an HFD.

## Materials and methods

### Animals and experiment protocol

Twenty male C57BL/6 J ApoE-KO mice and 10 healthy male C57BL/6 J mice were both purchased from the Beijing Vital River Laboratory Animal Technology Company (Beijing, China). All the mice were housed in microisolator cages and assayed under conditions of controlled temperature of 23–26 °C and humidity of 60% in a 12-h light/dark cycle with free access to standard chow and sterile water. After 1 week of acclimatization to the environment, twenty male C57BL/6 J ApoE-KO mice were randomly divided into two groups: High Fat Diet group (HFD group, *n* = 10) and High Fat Diet plus Exercise group (HFDE group, *n* = 10). These two groups were fed a high fat diet (D12451, 45% of total calories from fat, FBSH Biotechnologhy Inc., Shanghai, China) for 12 weeks, and meanwhile, ten healthy male C57BL/6 J mice received a standard diet (D12450J, 10% of total calories from fat, FBSH Biotechnologhy Inc.) were included in the Normal Diet group (ND group, *n* = 10). Furthermore, HFDE group underwent swimming training (i.e., 20 min on day 1, 30 min on day 2, 40 min on day 3, 50 min on day 4, 60 min on day 5 followed by 1 h per day for the next 11 weeks and continued the same protocol till week 12) in a round stainless steel water tank of 70 dm^3^ volume and 50 cm depth, with water temperature at 35 °C. Body weight of all the mice were monitored and recorded periodically. After 12 weeks, all the mice were sacrificed. Retro-orbital blood samples were collected for biochemical analysis. Liver tissues were immediately excised under sterile conditions followed by washing with ice-cold normal saline, and then stored at − 80 °C for further analysis.

### Biochemical analysis

Blood samples were collected from all the mice after overnight fasting. Fasting plasma glucose (FPG) was measured from tail vein blood by Glucose meter GM300 (Rightest Inc., Taiwan, China). Fasting serum insulin level (FIN) was assayed by INS ELISA Kit (Beijing North Institute, Beijing, China). Serum total cholesterol (TC), triglyceride (TG), high-density lipoprotein cholesterol (HDL), and FFA were measured by an automatic biochemical analyzer (HITACHI Company, Japan). Friedewald equation was applied to calculate LDL, with the following equation: LDL (mmol/L) = (TC-HDL-TG)/2.2. Homa-IRI was calculated to ascertain the establishment of IR, with the following equation: IRI = [FPG(mmol/L) × FIN(μIU/ mL)]/22.5).

### Western blot

The liver tissues were lysed on ice using RIPA lysis buffer (Cell Signaling Technology, USA) supplemented with protease inhibitors and phosphatase inhibitors (Roche, USA), and 1 mM PMSF (Sigma, USA). Equal amount of proteins were separated on 10% SDS-PAGE gels. The tissues were then transferred onto nitrocellulose membranes (Bio-Rad, USA), and were subsequently blocked with 5% skimmed milk. The membranes were incubated with primary antibodies against PPAR-γ (ab45036, Abcam, U.S.), CPT-1 (ab104662 Abcam, U.S.), MCAD (ab92461, Abcam, U.S.) and GAPDH (G9545, Sigma-Aldrich, U.S.). After washing with 0.1% Tween 20 in TBS, the membranes were incubated with horseradish peroxidase-conjugated secondary antibody (Santa Cruz Biotechnology, U.S.) and bound antibody was detected by ECL Plus Reagents (Amersham Biosciences, U.S.). Bands were quantified and analyzed by Image Pro Plus software.

### Histology examination

Liver samples were excised and embedded in Tissue-Tek OCT compound for histopathological analysis. The OCT-embedded samples were serially sectioned at 4 μm and stained with Oil Red O for the evaluation of fat deposition. To estimate adipogenesis of the liver, after removing the staining solution, the dye retained in the cells was eluted by 200 μL isopropanol, and absorbance was measured at 510 nm by microplate reader (Bio-RAD680, Bio-Rad Inc., USA) for quantitative analysis via Image Pro Plus software (Fig. 4).

### Statistical analysis

Statistical analysis was conducted using SPSS 16.0 statistical software. All the data were reported as means ± SD. One-way analysis of variance (ANOVA) was applied to determine statistical significance among different groups and Tukey’s test was used to evaluate the differences among the means of different treatments. *P* < 0.05 was considered statistically significant.

## Results

### Effect of exercise on body weight

**Fig. 1:**
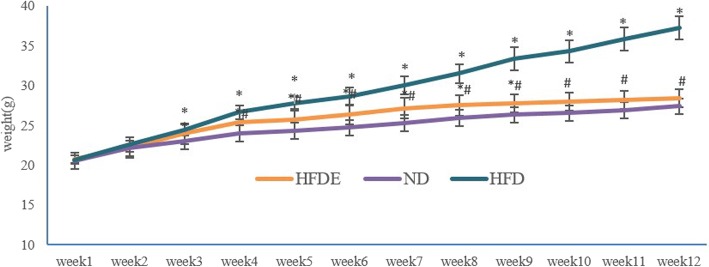
The weight change of three groups (Means±SD). The orange line marks the HFDE group, the purple line marks the ND group, and the blue line marks the HFD group. ^*^p < 0.05 VS ND group, ^#^*p* < 0.05 VS HFD group

At the beginning, there was no difference in body weight among the three groups of mice. After 12 weeks, compared with the ND group, body weight was significantly higher in the HFD group (37.25 ± 2.43 g VS 27.4 ± 0.52 g, *p* < 0.05). Furthermore, the body weight of the HFDE group significantly decreased after 12-weeks of swimming training compared with the HFD group (30.18 ± 1.10 g VS 37.25 ± 2.43 g; see Table 1, *p* < 0.05). The weight change trends of three groups were presented in Fig. 1**.**

**Table 1:** The weight change of three groups during 12 weeks (Means±SD, *n* = 30)

Time	Weight ND group (*n* = 10)	Weight HFD group (*n* = 10)	Weight HFDE group (*n* = 10)
Before	20.54 ± 0.73 g	20.69 ± 0.52 g	20.83 ± 0.48 g
After	27.40 ± 0.52 g	37.25 ± 1.49 g*	28.42 ± 1.10 g^#^

*p < 0.05 VS ND group, ^#^*p* < 0.05 VS HFD group

### Effect of exercise on the biochemical indicators

All the results were presented in Table 2. Results showed that the levels of TC, TG, LDL, FFA, FIN, FPG and Homa-IRI in the HFD group were significantly higher than the ND group (*p* < 0.05). However, after 12-weeks of intervention, the levels of TC, TG, LDL, FFA, FIN, FPG and Homa-IRI were markedly decreased in the HFDE group compared with the HFD group.

**Table 2:** Biochemical indicators of three groups after 12 weeks

Groups	TC	TG	HDL	LDL	FFA	FIN	FPG	IRI
	(mmol/l)	(mmol/l)	(mmol/l)	(mmol/l)	(mmol/l)	(mIU/ml)	(mmol/l)	
ND (*n* = 10)	4.78 ± 0.18	0.57 ± 0.11	2.13 ± 0.21	0.94 ± 0.23	1.00 ± 0.28	4.43 ± 1.30	5.58 ± 0.84	1.11 ± 0.17
HFD (*n* = 10)	20.82 ± 0.75*	1.12 ± 0.49^*^	1.52 ± 0.12	8.31 ± 0.44^**^	1.47 ± 0.27^*^	10.99 ± 0.82^**^	9.03 ± 1.17^**^	4.43 ± 0.43^**^
HFDE (*n* = 10)	12.95 ± 0.53#	0.67 ± 0.19^#^	2.11 ± 0.38	4.74 ± 0.35^##^	1.03 ± 0.20^#^	7.28 ± 0.75^#^	6.37 ± 0.71^#^	2.08 ± 0.47^##^

*TC* total serum cholesterol, *TG* triglyceride, *HDL* high-density lipoprotein cholesterol, *LDL* low-density lipoprotein cholesterol, *FFA* free fatty acid, *FIN* fasting serum insulin level, *FPG* fasting plasma glucose, *IRI* insulin resistance index. All the values are presented as Means ± SD. ^*^p < 0.05, ^**^ *p* < 0.01, compared with ND group; ^#^ *p* < 0.05, ^##^ *p* < 0.01, compared with HFD group

### Evaluation of liver histology

**Fig. 2:**
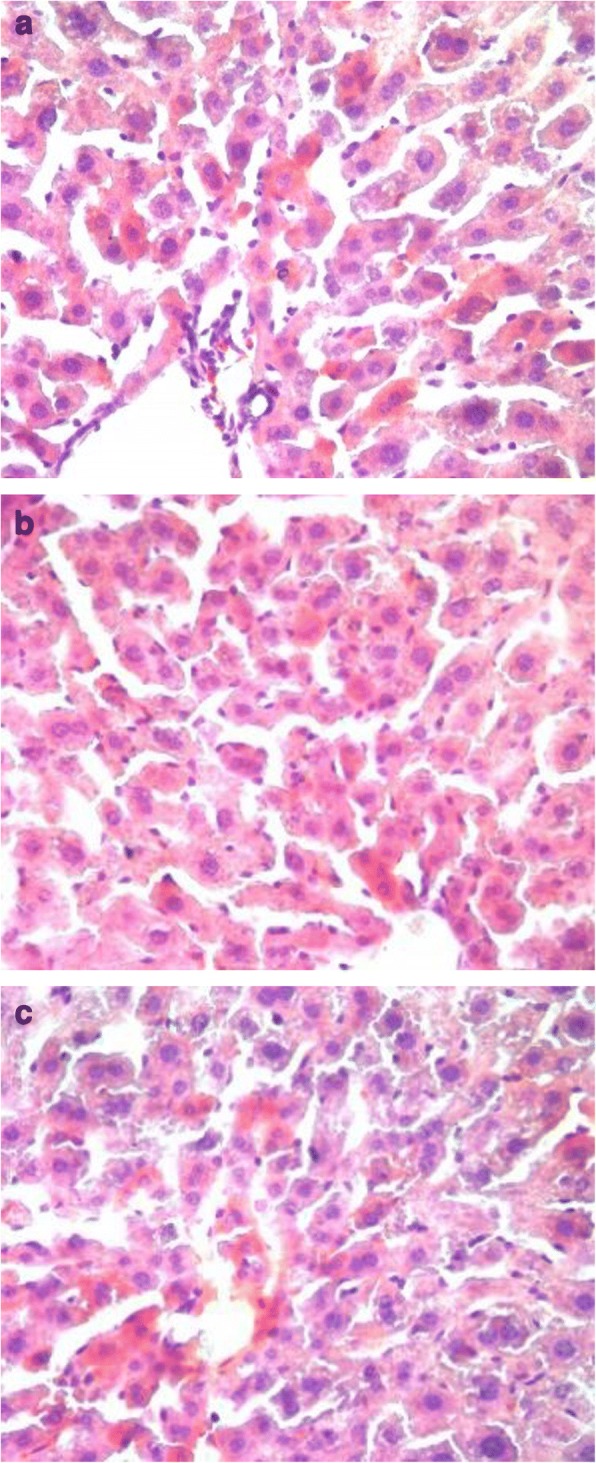
Oil Red O staining of liver sections in ND, HFD, and HFDE groups (400× magnification). **a** Oil Red O staining of ND group. **b** Oil Red O staining of HFD group. **c** Oil Red O staining of HFDE group

**Fig. 3:**
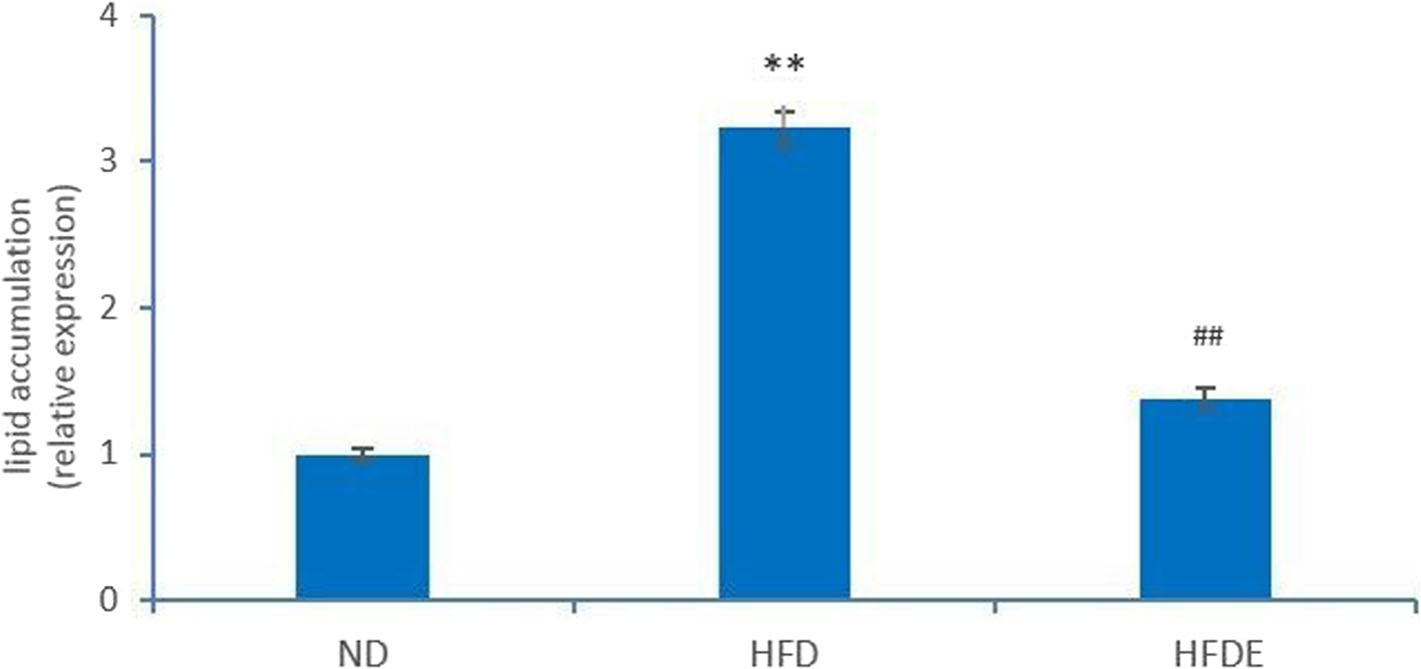
Quantified intracellular fat drops were extracted using isopropanol and measured by a microplate reader at 510 nm. The histogram shows the relative fold change compared with ND group (set as 1). Data are presented as Means±SD, *n* = 30. ** *p* < 0.01 vs ND group, ^##^*p* < 0.01 vs HFD group

Oil Red O staining of samples from the ND group occasionally showed scattered lipid drops without liver steatosis. Meanwhile, Oil Red O staining of samples from the HFD group showed severe fatty changes in the liver tissue and was were characterized by diffusely mixed sizes of fat bubble, but tended to have big fat bubbles. After 12 weeks of exercise intervention, the fatty proportion of the liver was significantly reduced in the HFDE group. Results showed a distinct increase in fat deposition in the liver tissue induced by an HFD, whereas swimming decreased the fat accumulation in the liver sections (Figs. 2 and 3).

### Expressions of PPAR-γ, CPT-1, and MCAD

**Fig. 4:**
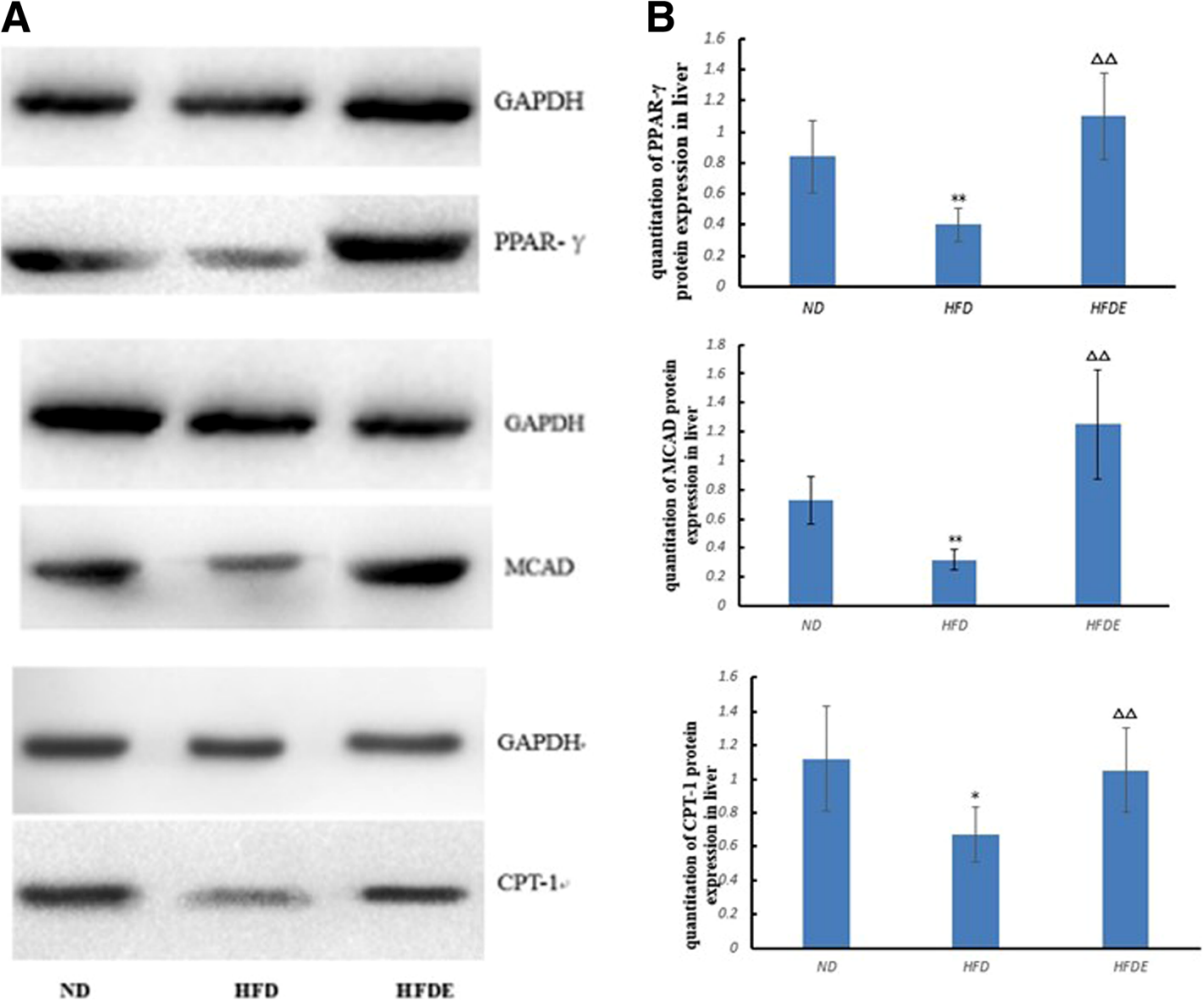
Effects of exercise on the protein levels of PPAR-γ, MCAD and CPT-1 in the livers of ApoE−/− mice fed an HFD. **a** Western blot bands of PPAR-γ, MCAD and CPT-1. **b** The relative protein expression levels were normalized by that of GAPDH (GAPDH, glyceraldehyde-3-phosphate dehydrogenase). *P < 0.05 vs. ND; ^ΔΔ^*P* < 0.01 vs. HFD

After 12 weeks of swimming exercise intervention, compared with the ND group, the protein levels of PPAR-γ, CPT-1 and MCAD in the HFD group were significantly decreased (*p* < 0.01). Meanwhile, compared with the HFD group, the protein levels of PPAR-γ, CPT-1 and MCAD in the HFDE group were significantly increased (*p* < 0.01; Fig. 4).

## Discussion

The first finding of this study was that the concurrent 12-week exercise could alleviate IR and counteract liver fat accumulation induced by an HFD. However, the evidence of exercise in the prevention of liver fat accumulation induced by an HFD still remains controversial. Some studies reported that exercise had no effect on liver lipid accumulation in HFD-induced obese rats [34, 35]. However, another study [36] demonstrated that concurrent exercise completely prevented hepatic steatosis induced by an HFD. The difference between these opposing findings might be owing to the fact that the latter study applied exercise at the same time as the induction of HFD, demonstrating that exercise should be conducted as early as possible to be effective.

On the other hand, the modalities of exercise treatment (i.e., exercise duration and exercise intensity) should also be considered to when evaluating its effects. Actually, there are no standard criteria for the modalities of an exercise regimen. It was reported that a concurrent 4-week exercise program failed to reverse hepatic steatosis induced by an HFD in rats [37]. This is probably because the exercise duration was too short to compensate for the metabolic disorder induced by the HFD. According to a recent study, a 12-week moderate aerobic exercise protocol proved to be effective in the management of T2DM and NAFLD progression in Otsuka Long-Evans Tokushima Fatty (OLETF) rats [38]. Our results were in accordance with the previous research [38], and showed that a moderate-intensity long-term exercise protocol was effective in suppressing fatty liver deposits and improving peripheral IR status. Notably, in the previously mentioned study, the exercise was started after the OLETF rats developed moderate NAFLD, so they could not completely reverse the disease condition. Thus, for it to have the greatest suppressive effect on IR and NAFLD, an exercise-regime should be applied as early as possible; also, the exercise should be at least a moderate intensity, and the regime should be adopted for the long term, as stated in the present study.

To gain insight into the concrete mechanism of how exercise prevents liver fat accumulation, we next measured the expressions of PPAR-γ, CPT-1, and MCAD in the livers of the three groups. As shown in the present study, the expressions of PPAR-γ, CPT-l, and MCAD in the HFDE group were increased, and the values of TC, FFA, and IRI were decreased as compared with the HFD group, indicating that exercise exerted anti-lipid accumulation effects through PPAR-γ / CPT-l / MCAD signaling. These results are in accordance with the previous study’s findings [39] that upregulation of PPAR evokes β-oxidation of fatty acids, increasing glucose uptake and catabolism, and in turn alleviates NALFD and improves IR. To our knowledge, most studies showed improved insulin sensitivity and lipid metabolism via PPAR-α target genes, CPT-1, and MCAD [40, 41], while few carried on with PPAR-γ / CPT-1/ MCAD signaling in the liver. This was partially done because PPAR-γ is mainly expressed in the adipose tissue, playing a vital role in the whole body lipid metabolism and insulin sensitivity [27]. However, under conditions of nonalcoholic fatty liver disease and nonalcoholic steatohepatitis, PPAR-γ was obviously overexpressed in the liver and correlated with insulin sensitivity [42, 43]. It was argued that the full activation of PPAR-γ triggered the transcription of lipogenic transcription factors, which favored obesity and NAFLD. However, the partial PPAR-γ activation could lead to an increased adiponectin level and insulin sensitivity, thus it would be a benefit to NAFLD and IR [44, 45]. It was reported that telmisartan, partially activated PPAR-γ combining with activating PPAR-alpha in the liver, could alleviate hepatic steatosis in mice that were fed an HFD [46]. In addition, telmisartan exerted beneficial effects on fatty livers in the treatment of human NAFLD when compared with losartan, highlighting the vital role of PPARs in the management of NAFLD [47]. Similarly, ragaglitazar, a dual PPAR-alpha/PPAR-gamma agonist, had beneficial effects on IR, hepatic steatosis and overweight induced by an HFD [48]. These drugs for NAFLD, including partially activated PPAR-γ, might mimic the effects of exercise on IR and hepatic steatosis. Moreover, evidence showed that increased PPAR-γ could upregulate CPT-1 and improve de novo lipogenesis and liver steatosis in rodents fed an HFD [27]. Based on the findings of the present study, we speculated that the expression of PPAR-γ was partially increased by long-term aerobic exercise, and it might concomitantly upregulate the expressions of CPT-1 and its target gene MCAD. This effect subsequently resulted in the alleviation of IR and NAFLD via enhanced fatty acid oxidation.

The highlight of this study is that the current study first provides insights on the effects of exercise on PPAR-γ down- regulation of CPT-1 and MCAD in the livers of mice on an HFD. This study provides potential targets for the clinical treatment of NAFLD in conjunction with IR. However, there are some limitations to this study. First, the sample size used in the study was determined by the sample size used by previous researchers [36, 49].. Due to the limited samples in this study, there is an increased risk of lower statistical power, and some important effects of physical training may not be detected. Second, we only involved male mice in the present study. However, a large body of evidence showed that NAFLD was a sexual dimorphic disease; that is, the male sex was at a higher risk of NAFLD [50]. Thus, the physiopathological peculiarities of NAFLD in females should be taken into account, and a more robust number of observations should allow for addressing the role of exercise for the two genders. Last, we only observed the expressions of PPAR-γ, CPT-1, and MCAD in the livers of mice with IR and NAFLD. Therefore, future research work should focus on the direct mechanism of exercise alleviation of IR and NAFLD in the liver tissue by regulating PPAR-γ/CPT-1/MCAD both in vivo and in vitro. The use of PPAR-γ knockout mice will clarify the exercise effects on IR and NAFLD via PPAR-γ/CPT-1/ MCAD signaling.

## Conclusions

Through a concurrent 12-week swimming training, lipid metabolism disorder and IR in the HFD ApoE-KO mice were significantly improved. The expressions of PPAR-γ, CPT, and MCAD in the HFDE group were significantly increased, suggesting that swimming training can partially activate the PPAR-γ expression in the liver tissue, concomitantly increase CPT-1 and MCAD levels, and finally alleviate the liver lipid disorders of IR and NAFLD.

## Abbreviations

ApoE-KO: Apolipoprotein E knock out
CPT-1: Carnitine palmitoyl transferase-1
FFA: Free fatty acid
FFAs: Free fatty acids concentrations
FIN: Fasting serum insulin level
FPG: Fasting plasma glucose
GAPDH: Glyceraldehyde-3-phosphate dehydrogenase
HDL: High-density lipoprotein cholesterol
HFD: High fat diet
HFDE: High fat diet plus exercise
IR: Insulin resistance
IRI: Insulin resistance index
LDL: Low-density lipoprotein cholesterol
MCAD: Medium-chain acyl-CoA dehydrogenase
NAFLD: Nonalcoholic fatty liver disease
NASH: Nonalcoholic steatohepatitis
ND: Normal diet
OLETF: Otsuka long-evans tokushima fatty
PPAR: Peroxisome proliferator-activated receptors
SD: Standard deviation
T2DM: Type 2 diabetes mellitus
TC: Total serum cholesterol
TG: Triglyceride
